# How RNA methylation regulates jasmonic acid biosynthesis and plant aging

**DOI:** 10.1093/plcell/koag323

**Published:** 2026-10-24

**Authors:** Annabelle Campbell

**Affiliations:** Assistant Features Editor, The Plant Cell, American Society of Plant Biologists; Department of Biology, Biological Sciences Bldg, 130 Science Dr, Duke University, Durham, NC 27708, United States

Post-transcriptional modifications impact all phases of an mRNA's lifespan, including splicing, transport, translation, and decay (Shen et al. 2023). The most prevalent modification of internal mRNA bases (ie not the 5′ or 3′ ends) is N^6^-methyladenosine, or m^6^A. This modification, and the complexes that “write” it, are highly conserved among eukaryotes. In *Arabidopsis thaliana*, the m^6^A writer complex consists of adenosine methylases A and B (MTA and MTB) together with the accessory proteins FIP37, VIR, and HAKAI (Ruzicka et al. 2017).

m^6^A drives many important developmental and stress responsive functions in the plant lifecycle, and different developmental stages display distinct patterns of m^6^A modifications (Zhang et al. 2024). Global m^6^A changes have been implicated in the hormonal control of development; specifically, MTA was shown to regulate jasmonic acid (JA) production and associated physiological processes, such as dark-induced leaf senescence (Sheikh et al. 2024). How this process is regulated, however, remained unclear.

It was previously reported that the protein HAKAI-INTERACTING ZINC FINGER PROTEIN 2 (HIZ2) plays an essential role in global m^6^A modification and proper organismal development; however, its exact function in the writer complex remained unknown (Zhang et al. 2022). In recent work, Yu Zhang and colleagues (2026) addressed this knowledge gap, demonstrating that HIZ2-mediated m^6^A regulates JA-driven leaf senescence in aging plants.

To determine the role of HIZ2 in the m^6^A writer complex, the authors began by characterizing *hiz2* mutant plants, which exhibit dysregulated development (eg delayed leaf initiation and growth) and reduced m^6^A levels, a phenotype shared among the other core component mutants. They then showed that HIZ2 directly interacts with the writer complex proteins, leading them to wonder whether HIZ2 regulates writer protein expression. Interestingly, core component transcript levels do not change in the absence of HIZ2, but their protein levels greatly decrease, suggesting HIZ2 plays a role in maintaining writer protein stability.

The authors then used nanopore sequencing to directly measure m^6^A methylation of RNAs in *hiz2* and wild-type plants, observing a dramatic decrease in m^6^A modification sites and target genes in the absence of HIZ2. They also evaluated the functions of specific gene classes which were both differentially expressed and methylated in the *hiz2* mutant. Notably, transcripts associated with JA biosynthesis were less methylated and more abundant in the absence of HIZ2.

Because active JA pathways are closely linked with promoting aging and leaf senescence, the authors looked for associated phenotypes in the *hiz2* mutant. Indeed, *hiz2* leaves showed visible yellowing during early development indicative of early senescence. Based on these observations, the authors speculate that in young wild-type plants, JA-associated transcripts are methylated by the m^6^A writer complex and become destabilized. As the plant ages, writer component expression decreases along with their methylation activity, JA-associated transcripts are stabilized, and leaf senescence progresses.

How then does m^6^A methylation destabilize transcripts? The authors looked for answers in the proteins that “read” RNA methylation. They found that the known m^6^A readers EVOLUTIONARILY CONSERVED C-TERMINAL REGION (ECT) 2/3/4 directly associate with both JA biosynthesis mRNAs and the protein XRN4, a key factor in cytoplasmic mRNA decay. Consistent with this route, the *ect2/3/4* triple mutant phenocopies *hiz2*, senescing early and over-accumulating JA-related transcripts, supporting the conclusion that these readers play a role in JA transcript destabilization.

The authors propose a model in which the m^6^A writer complex modifies JA-related mRNAs, leading to their recognition by ECT2/3/4 and destabilization via XRN4 (see Fig. 1). Writer expression decreases as the plant ages, resulting in reduced methylation and stabilization of JA-related genes, triggering leaf senescence. This work draws a novel connection between developmental signals and the phytohormone signaling that drives aging; however, no m^6^A -mediated regulation of other phytohormones like abscisic acid, salicylic acid, or auxin was observed. Most excitingly, m^6^A sequencing data from other species reveal JA biosynthesis genes are also modified in rice, tomato, and maize, indicating that this regulatory pathway is likely conserved among crop species.

**Figure 1:**
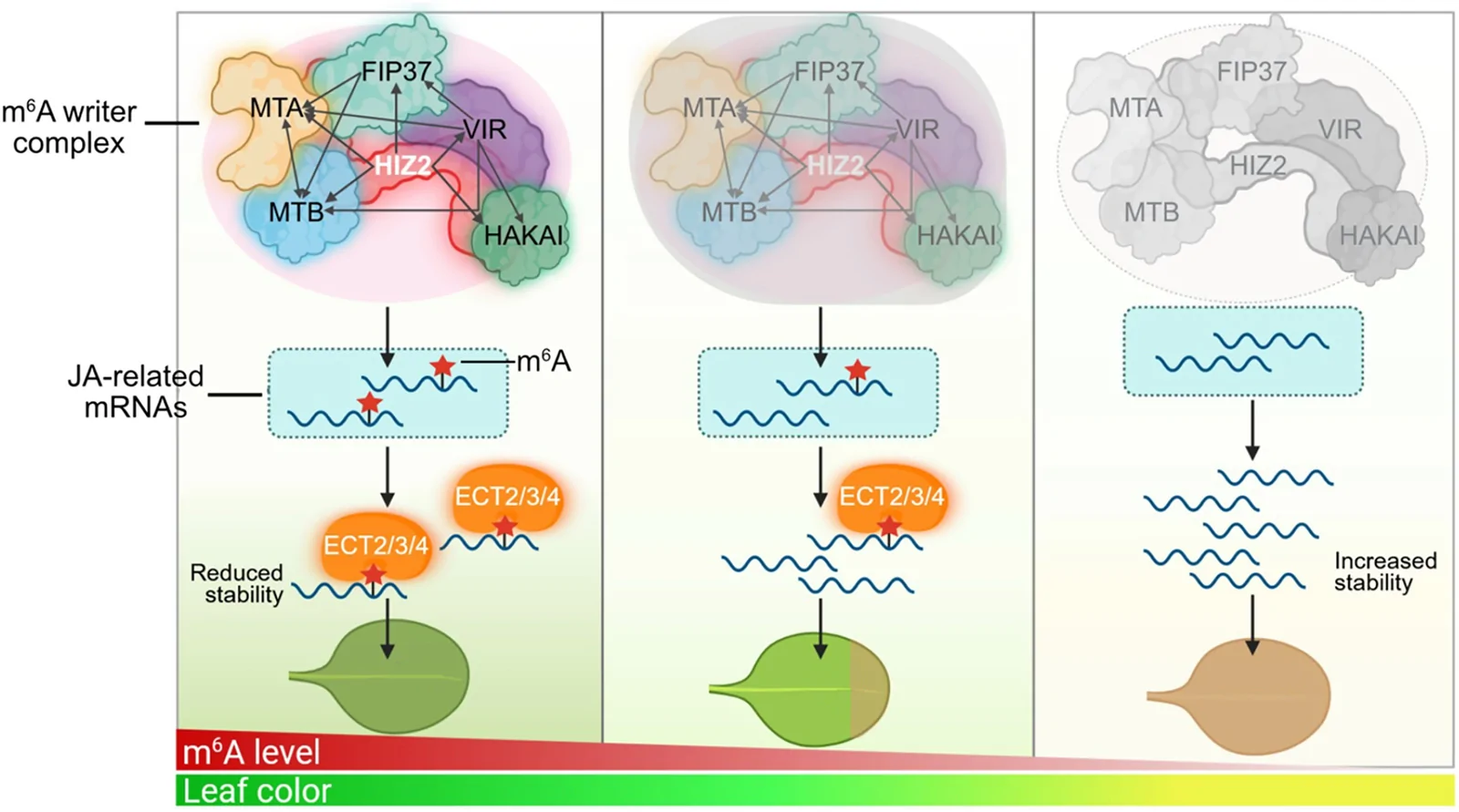
Model for m^6^A-mediated destabilization of JA mRNAs. In young leaves, the m^6^A writer complex is active, and JA-related mRNAs are methylated and targeted by the ECT2/3/4 reader complex for degradation (left panel). As the plant ages, the writer complex becomes less active and JA mRNAs are stabilized, promoting leaf senescence and its indicative color change (middle and right panels). Adapted from Zhang et al. 2026.

## Recent related articles in *The Plant Cell*

- Li et al. (2026) show that methylated RNA is bound by a reader protein to regulate aging in tomato leaves.
- Kang and Xu (2023) present an overview of recent developments in uncovering the role of m^6^A RNA methylation in liquid–liquid phase separation in plant cells.
- Prall et al. (2023) show that m^6^A RNA modification plays a vital role in the plant immune response by modulating transcript stability and abundance.

## Data Availability

The data underlying this article are available at https://doi.org/10.1093/plcell/koag287.
